# Over a century of pear breeding at the USDA

**DOI:** 10.3389/fpls.2024.1474143

**Published:** 2024-09-11

**Authors:** Christopher Gottschalk, Richard L. Bell, Gayle M. Volk, Chris Dardick

**Affiliations:** ^1^ Agricultural Research Service Appalachian Fruit Research Station, U.S. Department of Agriculture, Kearneysville, WV, United States; ^2^ Retired, Kearneysville, WV, United States; ^3^ Agricultural Research Service National Laboratory for Genetic Resources Preservation, U.S. Department of Agriculture, Fort Collins, CO, United States

**Keywords:** Pyrus (pears), history, fire blight (*Erwinia amylovora*), germplasm (genetic) resources, cultivar development

## Abstract

The U.S. Department of Agriculture (USDA) has performed European pear (*Pyrus communis* L.) scion breeding for over a century. The breeding program started in the early 1900s by Merton B. Waite in the Washington D.C. area and the program’s main goal was to develop host resistance to the devastating disease fire blight, caused by *Erwinia amylovora.* Most of the historic European pear cultivars being produced in the U.S. were susceptible to fire blight, prompting a need to breed for resistance. More than six generations of USDA breeders have continued this effort to breed disease-resistant European pears. Along with fire blight resistance, the pear breeding programs sought improved fruit quality, cold hardiness, and resistance to psylla (*Cacopsylla pyricola* Foërster), a significant insect pest of pear. Herein, we discuss the history of the program through each generation of breeder(s). We also present breeding aims, parental selection, and releases. In total, the program has released ten named pear varieties between 1938 and 2022.

## Introduction

The U.S. Department of Agriculture (USDA) has led breeding efforts for many crops, and it is particularly well positioned to breed long-term, perennials such as fruit trees because researchers receive base funding and are less dependent upon short-term grants. One of the longest running breeding programs in the USDA Agricultural Research Service (ARS) is the pear (*Pyrus* spp.) scion breeding program. The catalyst for the establishment of a pear breeding program in the U.S. was prompted by the lack of cultivars with resistance to fire blight caused by the bacterial pathogen *Erwinia amylovora* [(Burrill) Winslow et al.] (Waite, 1895; van der Zwet and Keil, 1979). *Erwinia amylovora* is believed to have originated in North America, thus, host resistance to this bacterial pathogen was lacking in much of the European germplasm that was brought to the U.S (van der Zwet and Keil, 1979; Bonn and van der Zwet, 2000). There was a broad effort to breed fire blight resistant pears across the U.S (van der Zwet and Keil, 1979). Although all the breeding programs had this shared goal, they were distinct in approach and germplasm utilized with many documented successes (van der Zwet and Keil, 1979). That said, improvements made by one program were ultimately shared with others. Unfortunately, many of the pear breeding programs ceased operations by 1970 due to various reasons such as closure of research stations and lack of consistent funding support (van der Zwet and Keil, 1979); however, the oldest continuous breeding program was initiated and continued to be supported by the USDA.

**Figure 1:**
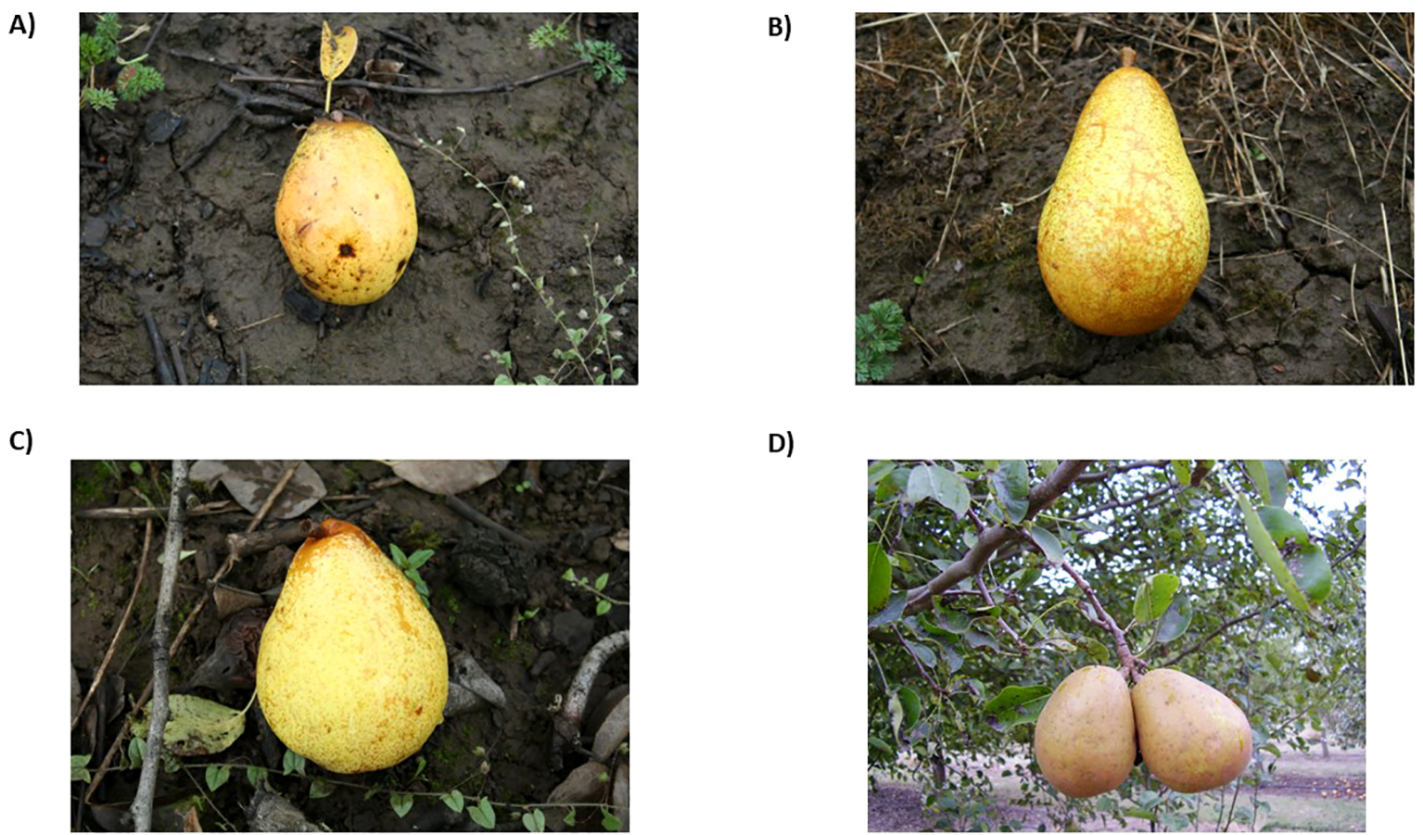
**(A)** ‘Waite’ (PI 66131) released in 1938. **(B)** ‘Dawn’ (PI 541170) released in 1960. **(C)** ‘Moonglow’ (PI 541315) released in 1960. **(D)** ‘Magness’ (PI 541299) released in 1968. Photos retrieved from GRIN-Global.

The USDA pear scion breeding program started in earnest in 1908, led by Special Agent Merton B. Waite at the Arlington Farm, Arlington, VA (Magness, 1937; van der Zwet and Keil, 1979). The Arlington Farm, once located on the southern bank of the Potomac River, no longer exists as the site was repurposed for the building of the Pentagon. Waite aimed to hybridize the moderately fire blight resistant ‘Kieffer’ with the susceptible cultivar ‘Bartlett’ and moderately resistant ‘Seckel’, and ‘Buerré d’Anjou’ to develop segregating populations (Magness, 1937; van der Zwet et al., 1974a; van der Zwet and Keil, 1979). This material was then screened for resistance using inoculation trials. Ultimately, a few fire blight resistant selections were identified. One selection believed to be ‘Kieffer’ × unknown was introduced as a new variety by Waite and aptly named ‘Waite’ (syn. ‘Canner’) (Magness, 1937; van der Zwet and Keil, 1979). ‘Waite’ is available from the USDA-ARS National Plant Germplasm System (NPGS) pear collection in Corvallis, OR, and is listed in the Germplasm Resources Information Network Global (GRIN-Global) information management system under PI 66131 (Postman et al., 2006; USDA Agricultural Research Service, 2024). ‘Waite’ is described as a large pear similar to ‘Bartlett’, with a yellow peel and smooth flesh, free of stone cells, but more acidic than ‘Bartlett’ (**Figure 1A**). It is not highly productive and, thus, was never widely planted as a fresh-market pear; however, it is recommended for cooking and canning (Brooks and Olmo, 1952; USDA Agricultural Research Service, 2024). Recent genotyping and identity-by-descent analysis found that ‘Waite’ is a cross of ‘Bartlett’ × ‘Buerré d’Anjou’, which could explain its similarity to its parents (Montanari et al., 2020) (**Supplementary Figure 1**).

Between 1916 and 1919, a cooperative breeding program between USDA and the Michigan Agricultural Experiment Station (established by Michigan Agriculture College, now known as Michigan State University) in South Haven, MI was initiated (Magness, 1937; van der Zwet and Keil, 1979). It was led by USDA Botanists William F. Wight and Don Ward along with graduate student Stanley Johnston (later a Michigan State University professor) (Magness, 1937; van der Zwet and Keil, 1979). The aim of this program was to intercross pear germplasm with moderate fire blight resistance with high fruit quality pears. The most impactful crosses were generated from seedlings of ‘Barseck’ ([‘Bartlett’ × ‘Seckel’] × ‘Barlett’). The breeding line Mich-US 437, believed to be ‘Barseck’ × ‘Barlett’ was produced and was featured prominently as a parent in the later USDA breeding program (van der Zwet and Keil, 1979). As with ‘Waite’, genotyping analyses found that Mich-US 437 is actually ‘Vermont Beauty’ × ‘Barlett’ (Montanari et al., 2020) (**Supplementary Figure 2**), with ‘Vermont Beauty’ a descendant of ‘Seckel’ and ‘White Doyenné’, a parent of ‘Bartlett’ (Montanari et al., 2020). In the 1920s, W.F. Wight, who was leading the cooperative program in Michigan, moved to Palo Alto, California (Magness, 1937; The Stanford Daily, 1954). While in California, Wight continued to breed pears on behalf of the USDA-ARS while also joining Stanford University as a faculty member (Magness, 1937; The Stanford Daily, 1954). He aimed to develop high quality dessert pears, new summer pears with different harvesting intervals, and storage varieties of winter pears. The crosses by Wight were primarily crosses among a few *P. communis* varieties (Magness, 1937).

In 1938, John R. Magness led the USDA pear breeding program that had been moved from Arlington, VA to Beltsville, MD (van der Zwet and Keil, 1979). Under the direction of Magness, the focus of the pear breeding program continued to be the development of fire blight resistant cultivars (Magness, 1937). Magness recognized that many of the most resistant material was generated from interspecific hybridization of *P. communis* × *P. pyrifolia* (syn. *P. serotina*) but they lacked the quality of *P. communis* fruit cultivars. Magness also noted the need for improved cold hardiness for the Northern Great Plains production areas, the development of late ripening, and storage varieties for the West Coast regions (Magness, 1937). Magness also used new materials from the other pear breeding programs throughout the U.S. Moderately fire blight resistant European *P. communis* varieties such as ‘Roi Charles de Wurtemberg’ and ‘Doyenné du Comice’ (syn. ‘Comice’) were hybridized with breeding lines and three varieties were released (van der Zwet and Keil, 1979). In 1960, ‘Dawn’ (PI 541170; Mich-US 437 × ‘Doyenné du Comice’) and ‘Moonglow’ (PI 541315; Mich-US 437 × ‘Roi Charles de Wurtemberg’) were released (**Supplementary Figures 3**, **4**). ‘Dawn’ is an upright vigorous tree with sweet, aromatic, juicy yellow colored pear with an early ripening date (**Figure 1B**). ‘Dawn’ is moderately resistant to fire blight, similar to its parent ‘Doyenné du Comice’ (van der Zwet et al., 1974a). ‘Moonglow’ is a vigorous, productive, and precocious tree with mild flavored, yellow fruit with an early ripening date, and strong fire blight resistance (**Figure 1C**) (van der Zwet et al., 1974a). In 1968, ‘Magness’ was released and named in honor of J.R. Magness (PI 541299). ‘Magness’ resulted from a ‘Giant Seckel’ × ‘Doyenné du Comice’ cross (Montanari et al., 2020) (**Supplementary Figure 5**). ‘Magness’ is a very vigorous tree with russeted fruit that is sweet, highly juicy, and aromatic (**Figure 1D**). It also exhibits strong fire blight resistance (van der Zwet et al., 1974a). However, that resistance is tissue specific with the trunk being specifically susceptible (van der Zwet and Keil, 1970, 1972). The flavor of ‘Magness’ is similar to its parent ‘Comice’. Of note, ‘Magness’ is pollen sterile.

In 1960, the breeding program was transferred from J.R. Magness to Howard J. Brooks and Tom van der Zwet (van der Zwet and Keil, 1979). Under Brooks and van der Zwet’s leadership, the USDA pear breeding program undertook a more expansive direction. They set five objectives: 1) acquisition of new germplasm from around the world for evaluation of fire blight resistance, 2) evaluate the use of Asian pear species as parents for fire blight resistance, 3) mass seedling evaluations using a 10-year rotational schedule (trees selected or removed in 10 years), 4) greenhouse inoculation trials to screen for fire blight resistance, 5) investigate genetics that underlie fire blight resistance, tree juvenility, and fruit quality traits (van der Zwet and Keil, 1979). Additionally, the breeding program developed a cooperation with the Ohio Agriculture Research and Development Center at Wooster, OH which was led by W.A. Oitto and Roland C. Blake (van der Zwet and Keil, 1979).

Many Asian *Pyrus* species were collected in the early 1900s and in the 1970s this diversity was expanded through van der Zwet’s germplasm explorations to Eastern Europe to source hundreds of new *P. communis* and interspecific hybrids (van der Zwet et al., 1983, 1987, 1989; Waite et al., 2024). These materials were screened for fire blight resistance in the early 1970s (van der Zwet et al., 1974b). *P. ussuriensis* Group I plants were found to have the highest resistance to fire blight in the species category (van der Zwet et al., 1974b). One of the most impactful discoveries in this screening was that ‘Illinois 76’, a *Pyrus ussuriensis* × *P. pyrifolia* hybrid, produced a number of progeny that were highly resistant to fire blight (van der Zwet et al., 1974b). ‘Illinois 76’ was a selection that originated from the Rutgers University pear breeding program. Moreover, van der Zwet’s team developed useful protocols for mass seedling resistance screening and rating systems (Oitto et al., 1970; Van der Zwet and Oitto, 1973). This team was prolific, generating more than 42,000 seedlings from 600 crosses (van der Zwet and Keil, 1979). Although productive, the USDA program failed to release new varieties since ‘Magness’ in 1968.

In 1979, the USDA-ARS opened the Appalachian Fruit Research Station (AFRS) in Kearneysville, WV and the pear breeding program transferred to the new location. With the move to AFRS, the pear breeding program leadership was transferred to Richard L. Bell and T. van der Zwet. Their primary goals were to 1) develop additional cultivars with fire blight resistance combined with high fruit quality, long storage life, precocity and high productivity, 2) identify sources of resistance to *Diplocarpon mespili* (Sorauer) B. Sutton, which causes *Fabraea* leaf spot, and incorporate that resistance into breeding populations, selections, and cultivars, and 3) develop cultivars with resistance to pear psylla.

Research on resistance to pear psylla included the determination of the major pear psylla behavioral traits associated with resistance, the use of specific resistance assays to identify resistant germplasm and study the inheritance of these modes of resistance (Bell and Puterka, 2004). In this research, Drs. Bill Butt and Gary Puterka played major roles. Modes of pear psylla resistance were identified to be ovipositional antixenosis, antibiosis (*i.e.*, mortality), decreased development rate, and fecundity of surviving adult females. Assays characterized the resistance or susceptibility in *P. ussuriensis* hybrid selections and Eastern European germplasm (Butt et al., 1988, 1989; Bell, 1991, 1992, 2003, 2013; Puterka, 1997). Germplasm utilized included hybrid selections from the Cornell University and Rutgers University pear breeding programs. Several hybridizations generated seedling populations for evaluation, inheritance studies and cultivar development. Fruit quality and other traits of *P. ussuriensis* hybrids and Eastern European germplasm were evaluated (Bell, 2014).

**Figure 2:**
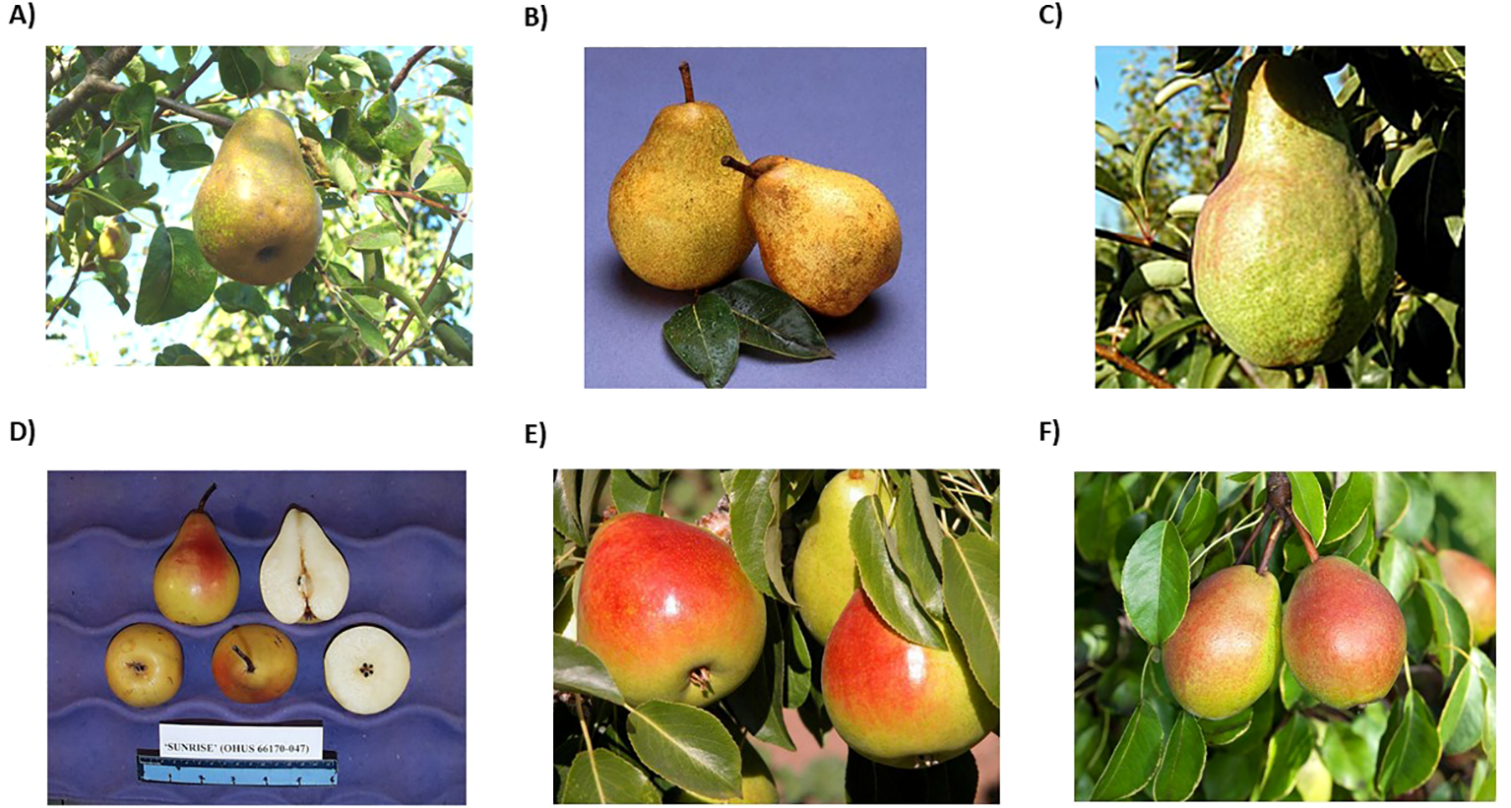
**(A)** ‘Potomac’ (PI 617594) released in 1993. **(B)** ‘Blake’s Pride’ (PI 657922) released in 1998. **(C)** ‘Shenandoah’ (PI 665743) released in 2003. **(D)** ‘Sunrise’ (PI 665744) released in 2006. **(E)** ‘Gem’ (PI 688121) released in 2013. Photo retrieved from Bell et al., 2014. **(F)** ‘Bell’ released in 2022, photo by Peggy Grub. All other photos retrieved from GRIN-Global and USDA-ARS.

Under Bell’s leadership, the USDA breeding program was highly productive, with five released varieties. The first release was ‘Potomac’ (PI 617594; ‘Moonglow’ × ‘Buerré d’Anjou’) in 1993. ‘Potomac’ is a moderately vigorous tree with medium-size green fruit similar in favor to ‘Beurré d’Anjou’ and strong fire blight resistance (**Figure 2A**) (Bell et al., 1996). ‘Blake’s Pride’ (PI 657922; US 446 × US 505) was released in 1998 (Bell et al., 2002). Named after R.C. Blake, ‘Blake’s Pride’ is an upright, moderately vigorous tree with juicy, russeted yellow fruit. ‘Blake’s Pride’ exhibits strong fire blight resistance and moderate resistance to pear scab (**Figure 2B**). Next was ‘Shenandoah’ (PI 665743; ‘Max Red Bartlett’ × US 56112-146 [US 309 open-pollinated]) in 2003. ‘Shenandoah’ is a moderately vigorous tree, with sweet aromatic, yellow-green fruit harvested late in the season, with moderate fire blight resistance (**Figure 2C**) (Bell and van der Zwet, 2008). ‘Shenandoah’s’ fruit flavor is acidic upon harvest but mellows during cold storage. Following ‘Shenandoah’ was ‘Sunrise’ (PI 665744; NJ 5001710820 × US 446) in 2006 (**Figure 2D**) (Bell and van der Zwet, 2011). ‘Sunrise’ represents the first release that has *P. pyrifolia* in its pedigree (NJ 5001710820 is ‘Bartlett’ × NJ 1 [open pollinated *P. pyrifolia*]). ‘Sunrise’ is a precocious, upright, moderately vigorous tree with large yellow fruit that is moderately juicy, sweet and aromatic. ‘Sunrise’ is very resistant to fire blight and moderately resistant to pear scab, and the fruit has a long shelf life (Bell and van der Zwet, 2011). The last release by R.L. Bell and T. van der Zwet was ‘Gem’ (PI 688121; ‘Sheldon’ × US 62563-004) in 2013 with numerous university partners (**Figure 2E**) (Bell et al., 2014). ‘Gem’ is a precocious, productive, tree with very attractive fruit with excellent fruit qualities and storage potential. ‘Gem’ can be eaten immediately after harvest for a crisp texture, or stored for up to five months, ripened, and eaten with a melting flesh texture. ‘Gem’ is susceptible to fire blight but is more resistant than ‘Bartlett’ (Bell et al., 2014).

## Current status and prospectus

The USDA pear breeding program underwent a short hiatus from 2017 until 2021 following the retirement of R.L. Bell and on-boarding of the new breeding program leader Chris Gottschalk. In 2022, ‘Bell’ (‘Lucious’ × US 65003-023) was released in collaboration with Pennsylvania State University (**Figure 2F**) (Bell et al., 2023). ‘Bell’, named after R.L. Bell, is a vigorous tree with excellent fruit quality. The fruit are harvested early and ripen on the tree. ‘Bell’ fruit have a melting texture with high sugar and high acidity, resulting in a highly desirable flavor. ‘Bell’ is also highly resistant to fire blight (Bell et al., 2023). ‘Bell’ is well positioned for direct-to-market opportunities, due to its unique fruit quality traits and limited storage potential.

The current USDA-ARS pear scion breeding program has two project aims: 1) development of segregating mapping populations to enable mapping of important traits and 2) evaluation and release of superior pear varieties. Under the first aim, there are three main objectives. First, develop populations to target high-value traits. Currently, target traits include fruit quality traits such as acidity, polyphenolics, and sugar content, post-harvest disease and disorder resistance, novelty such as red-flesh and perry (fermented pear juice) traits, and production efficiency such as semi-dwarf and columnar growth habits. The second objective is to generate new genomic resources such as whole genome sequencing along with genotyping tools to enable accurate and fine mapping of trait genetic determinants. Under the second aim, there are two objectives: 1) to evaluate existing USDA-ARS breeding program germplasm for advanced trials and release and 2) to develop new germplasm that combines high-value and industry disruptive traits. As a result of the long-term efforts of the previous USDA breeding programs, the current program is well-positioned to release cultivars with both disease resistance and high fruit quality.

## Conclusion

Even after over a century of breeding new pear varieties, there hasn’t yet been wide acceptance of new pear varieties in the U.S. Currently, 96% of the pear production is made up of four historic (>100 year old) varieties (USA Pears, 2024; Waite et al., 2024). Those four varieties are ‘Bartlett’, ‘Beurré d’Anjou’, ‘Beurré Bosc’, and ‘Red Anjou’ (USA Pears, 2024; Waite et al., 2024). Why haven’t these four varieties been supplanted by a modern release? The answer can be attributed to numerous factors such as the pear industry moving from the East Coast to the Pacific Northwest where disease pressure is lower due to the dry climate, challenges associated with complex storage and ripening requirements, stagnant productivity due to the lack of a dwarfing rootstock, and consumer acceptance. The USDA-ARS Pear Breeding Program is increasingly having an impact, demonstrated by the Pacific Northwest pear industry’s growing interest in ‘Gem’ since its release in 2013 (Odegard, 2024). Furthermore, the pear industry has expressed interest in testing other new varieties and exploring alternative marketing opportunities (*e.g.*, direct-to-consumer), climate change threats are increasing needs for abiotic and biotic resistance in production regions that traditionally were not challenged by these factors, and consumer interest in trying new varieties of tree fruit has reshaped the marketplace (*e.g.*, ‘Honeycrisp’ apple) (Odegard, 2024; Waite et al., 2024). The USDA-ARS Pear Breeding Program will continue to support the U.S. pear industry by delivering new varieties with traits that target these emerging industry needs.

## Germplasm availability

All released varieties from 1938 through 2013 (‘Waite’ through ‘Gem’) are available for research, breeding, and education from the USDA-ARS pear collection. Efforts are ongoing to ensure that remaining varieties developed by the program (*e.g.*, ‘Bell’) and vital genetic resources such as founder varieties and historic breeding lines (*e.g.*, Mich-US 437 and Illinois 76) are made publicly available through the NPGS.

## Data Availability

The original contributions presented in the study are included in the article/**Supplementary Material**. Further inquiries can be directed to the corresponding author.
