# Supplementary figures and images for: Over a century of pear breeding at the USDA
Source: Front Plant Sci. 2024 Sep 11;15:1474143. doi: 10.3389/fpls.2024.1474143 (PMC11422061; doi:10.3389/fpls.2024.1474143)

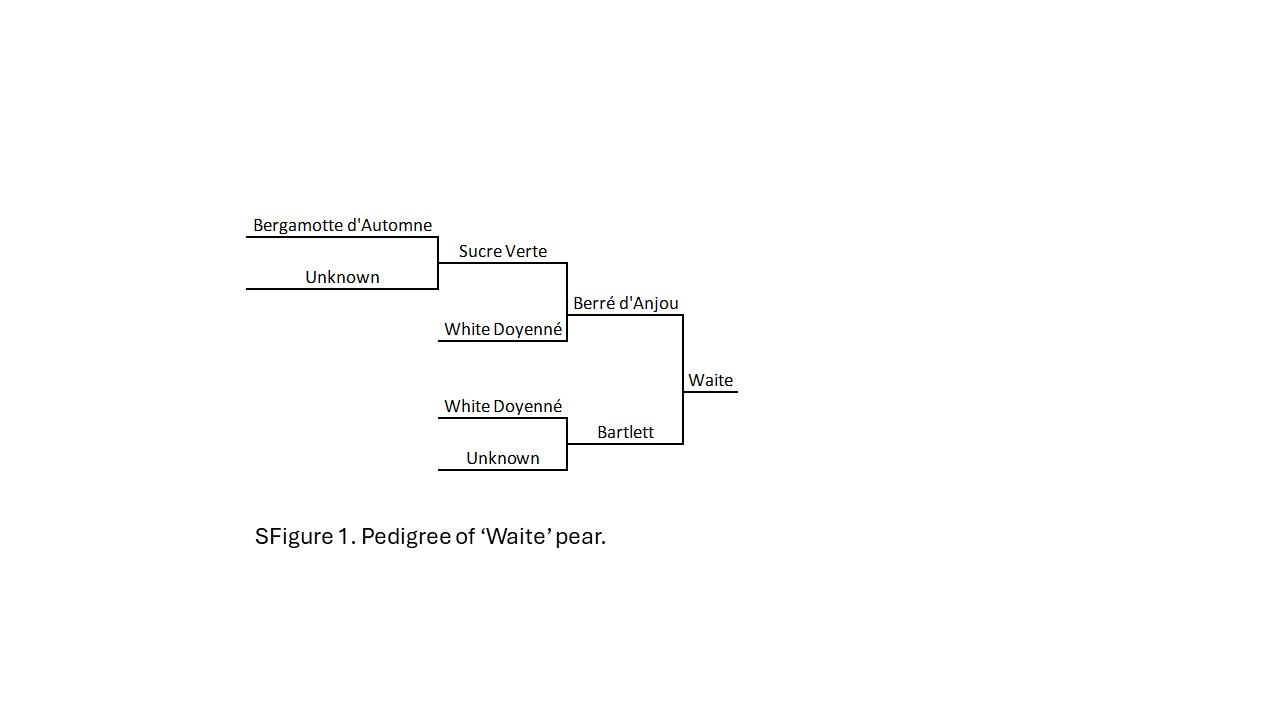

Supplement: Supplementary file 1 [file Image1.jpeg]

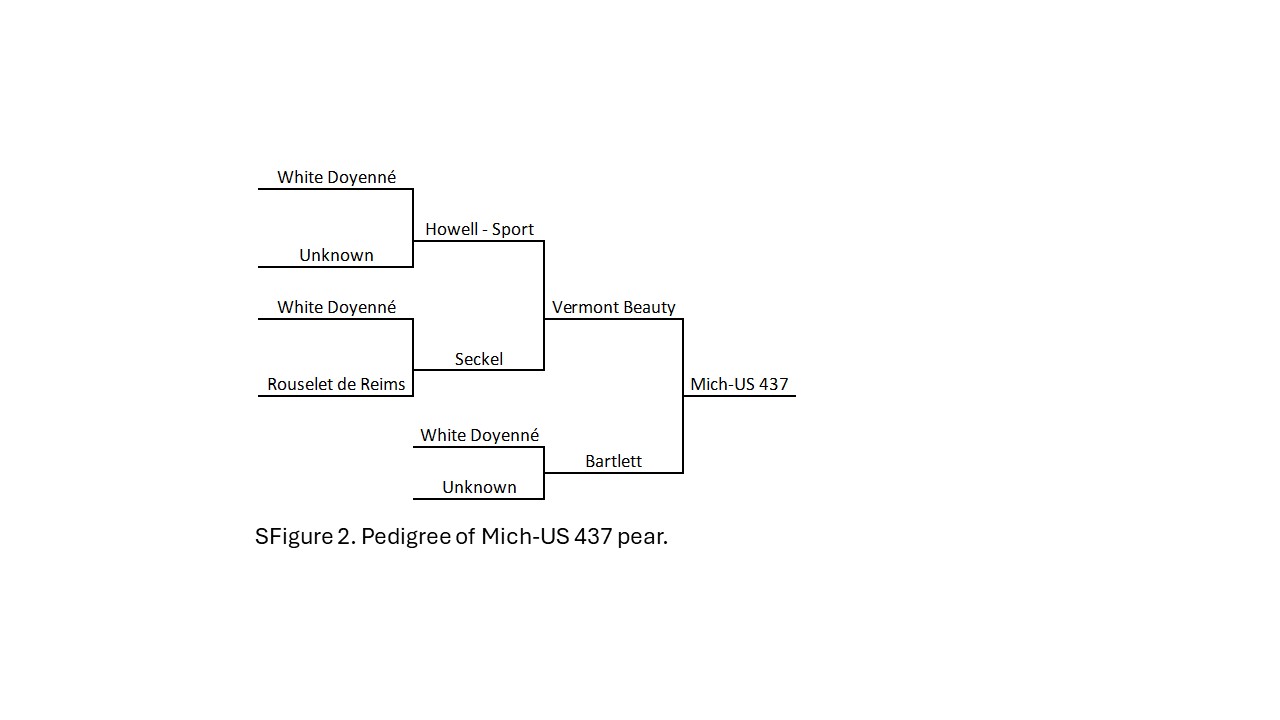

Supplement: Supplementary file 2 [file Image2.jpeg]

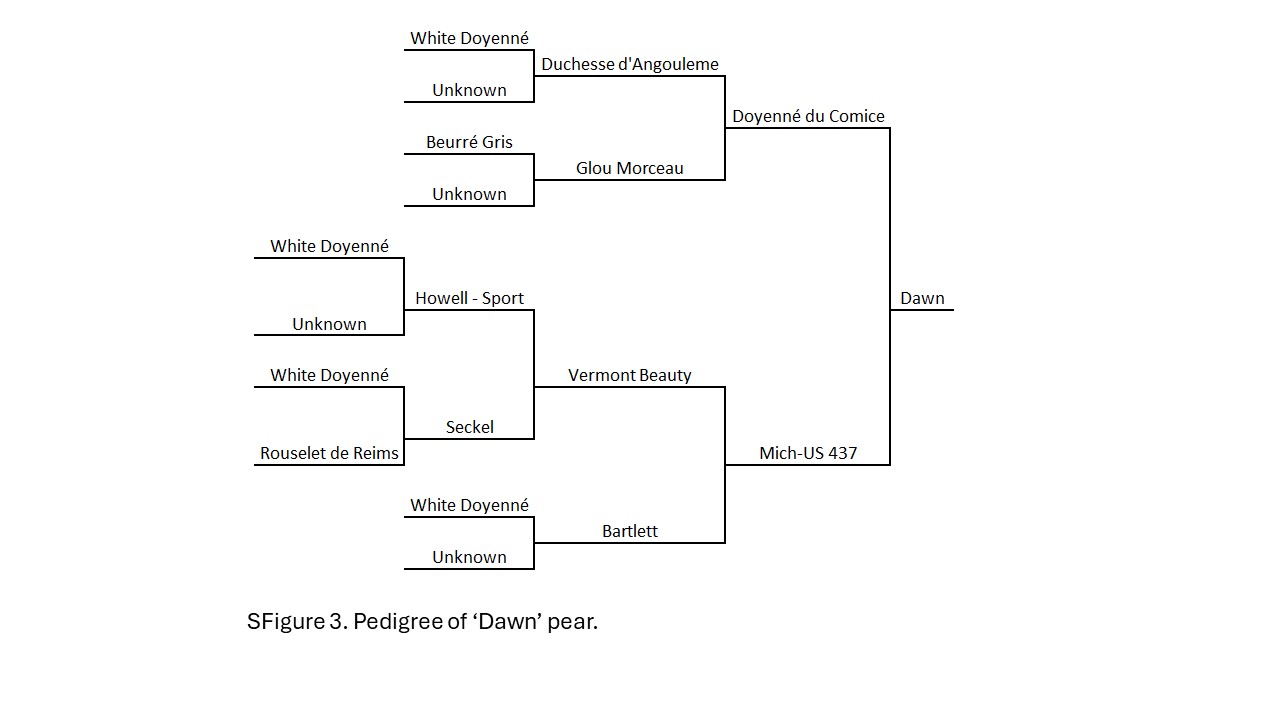

Supplement: Supplementary file 3 [file Image3.jpeg]

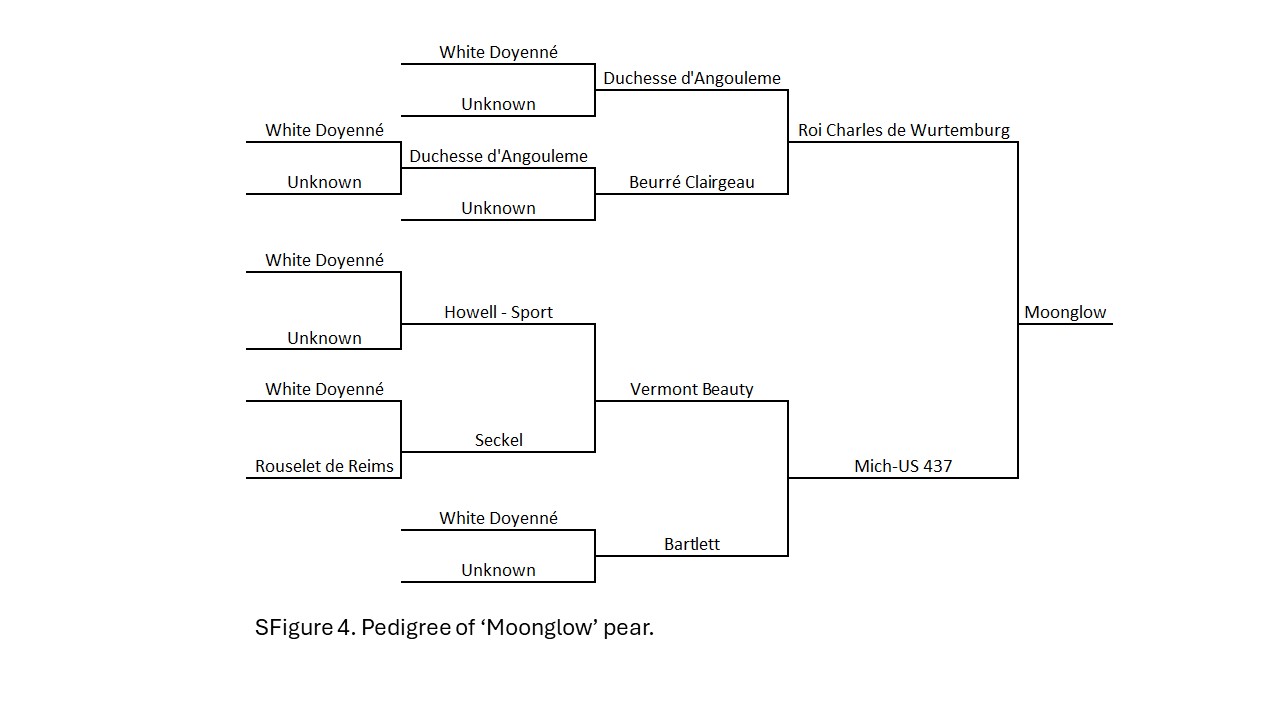

Supplement: Supplementary file 4 [file Image4.jpeg]

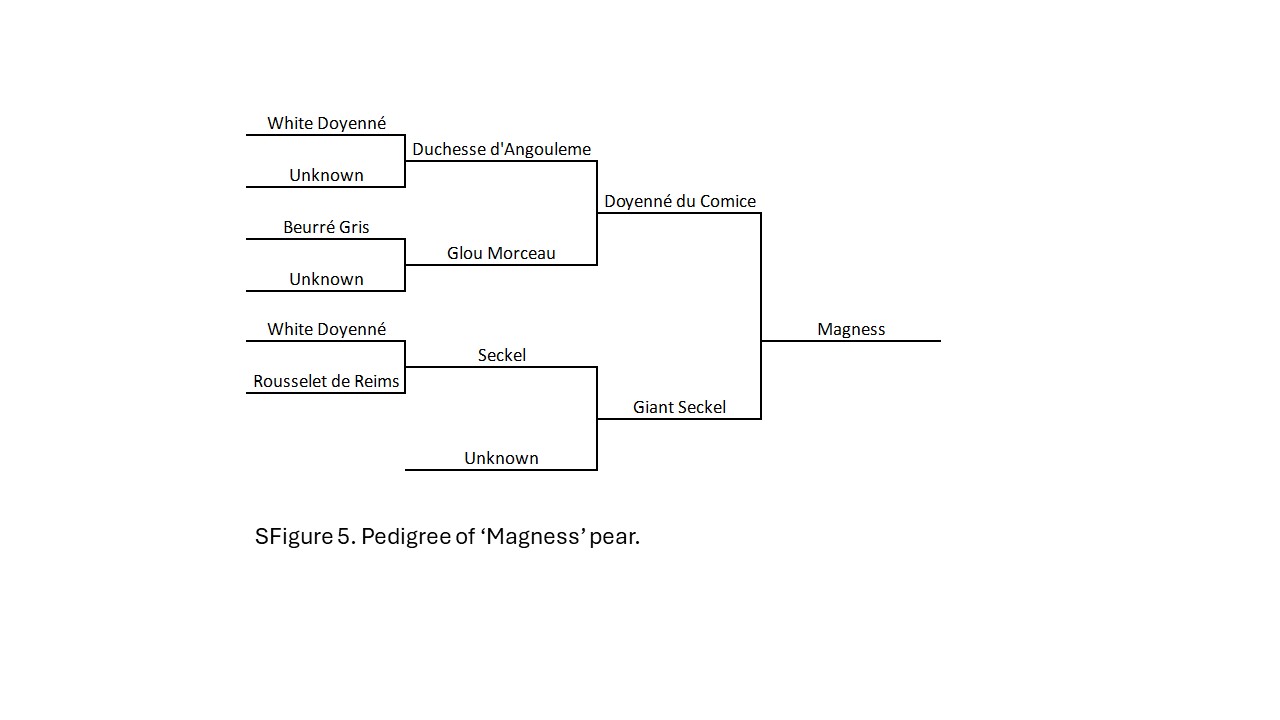

Supplement: Supplementary file 5 [file Image5.jpeg]
